# Quantitative evaluation of anti-sliding performance and dosage regulation of steel slag asphalt mixture facing service environment

**DOI:** 10.1371/journal.pone.0352143

**Published:** 2026-06-18

**Authors:** Zhiqiao Cheng, Ping Li, Jingsheng Pan

**Affiliations:** 1 Department of Civil Engineering, Chengdu Technological University, Yibin, ‌‌China; 2 School of Highway, Chang’an University, Xi’an, Shaanxi, ‌‌China; Shandong University of Technology, CHINA

## Abstract

In view of the practical demands for quantitative evaluation on skid resistance and scientific regulation of steel slag content of steel slag asphalt mixtures under service conditions, this study takes steel slag content, temperature and abrasion effect as research factors. Via accelerated loading abrasion tests, this paper investigates the evolution characteristics and quantitative evaluation models of two core skid resistance indicators namely British Pendulum Number (BPN) and Mean Texture Depth (MTD) under three steel slag contents and three temperature gradients. The results show that both BPN and MTD values of steel slag asphalt mixtures decrease obviously with the increase of abrasion cycles. High temperature can significantly accelerate the deterioration of skid resistance, which provides important experimental references for the optimization and regulation of steel slag content in service areas with different temperatures. BPN and MPN present generally high correlation under various temperatures and steel slag contents, which verifies the coordinated variation rule of the two skid resistance indicators under multi-factor conditions. From the perspective of staged abrasion degradation, the abrasion evolution processes of BPN and MTD can be clearly divided into low, medium and high abrasion stages. Their correlations differ greatly in different stages, laying a solid foundation for segmented prediction of skid resistance service life. The research findings further improve the quantitative evaluation system for skid resistance of steel slag asphalt mixtures oriented to actual service environment, and offer accurate data support and practical guidance for scientific adjustment of steel slag content in practical engineering.

## 1. Introduction

With the rapid development of highway transportation in China, the importance of pavement skid resistance to driving safety has become increasingly prominent [1,2]. Insufficient skid resistance of pavement is particularly likely to cause traffic accidents under complex conditions such as ice and snow [3–6], rainfall [7–10], and the existence of attachments or contaminants on the pavement surface [10,11]. Therefore, the development of asphalt mixtures with excellent skid resistance and good durability has become one of the research hotspots in the field of road engineering. As a large-scale solid waste from the iron and steel industry, steel slag is characterized by high hardness, good angularity and rough surface. Applying steel slag as aggregate in asphalt mixtures can not only effectively improve the skid resistance of pavement, but also realize the resource utilization of steel slag, which conforms to the development concepts of green transportation and circular economy [12–14].

To improve pavement skid resistance, studies worldwide have carried out systematic exploration from macro-texture, micro-texture, material properties and friction mechanism. It is generally accepted in existing studies that pavement surface friction performance consists of macro-texture depth and micro-texture. The former mainly affects low-speed friction and drainage performance, while the latter determines friction stability under high-speed conditions [15,16]. In terms of materials, aggregates with high strength and strong angularity have been proved to effectively enhance the retention capacity of pavement texture [13,17,18]. Early studies mostly focused on the anti-polishing characteristics of natural aggregates such as basalt, limestone and granite. In recent years, steel slag has been regarded as an ideal alternative material for improving the skid resistance of surface layers due to its high hardness, porous structure and superior abrasion resistance [19,20]. Steel slag has significant advantages in improving the abrasion resistance of mixtures, enhancing the retention of surface roughness and suppressing texture degradation, and can maintain high friction performance in wet and slippery environments [21,22]. In addition, some studies have attempted to reveal the texture evolution mechanism of steel slag from a microscopic perspective. For example, the pore structure on the steel slag surface is gradually exposed during the abrasion process, forming a dynamic thickening effect that helps maintain long-term skid resistance [23,24]. However, studies on the friction behavior of steel slag asphalt mixtures under different temperature environments are insufficient, and existing models cannot fully reflect the influence of temperature on texture crushing, pore exposure and aggregate-asphalt interface behavior. Moreover, most abrasion tests only focus on the single index of friction coefficient and lack correlation models among texture depth, texture degradation rate and abrasion loss, leading to great uncertainty in performance prediction of steel slag pavement. In general, although existing studies have proved that steel slag has good skid resistance potential, the formation mechanism, degradation law and environmental sensitivity of its skid resistance have not been systematically clarified.

At present, most existing studies on the skid resistance of steel slag asphalt mixtures mainly focus on the influence of single factors such as steel slag content, temperature or abrasion on skid resistance indicators. There is still a lack of systematic research on the evolution law of skid resistance under the combined effect of steel slag content, ambient temperature and accelerated abrasion. Pavement skid resistance is mainly characterized by British Pendulum Number (BPN) and Mean Texture Depth (MTD). BPN reflects the friction resistance between pavement surface and tires, while MTD represents the macro-texture characteristics of pavement surface. The joint effect of the two indicators determines the overall pavement skid resistance performance [25–27]. In practical engineering, steel slag asphalt pavements are simultaneously affected by different steel slag contents, seasonal temperature changes and long-term traffic abrasion. Accordingly, this paper takes steel slag asphalt mixtures as the research object. Based on accelerated loading abrasion tests, this study systematically explores the variation rules of BPN and MTD under different abrasion cycles at three steel slag contents of 0%, 50% and 100% and three ambient temperatures of 20°C, 40°C and 60°C, and reveals the influence mechanism of steel slag content, temperature and abrasion on its skid resistance.

## 2. Materials and methods

### 2.1. Materials

The coarse aggregate used in this study is basalt, and its technical properties are

shown in Table 1. The asphalt used is SBS-modified asphalt, and its technical properties are presented in Table 2.

**Table 1:** Technical Indicators of Coarse Aggregate.

Technical Index	Test Result	Technical Requirement	Test Method
**Crushing value/ %**	17.1	≤26	T0316
**Los Angeles abrasion value/ %**	15.1	≤28	T0317
**Polished Stone Value (PSV)**	63	≥42	T0321
**Flat and elongated particles/ %**	8.1	≤15	T0312
**Adhesion with asphalt (grade)**	5	≥4	T0616

**Table 2:** Asphalt Technical Properties.

Technical Index	Penetration (25 °C, 100 g, 5 s)/ 0.1 mm	Softening Point (Ring-and-Ball Method)/ °C	Ductility (5 cm/min, 5 °C)/ cm
**Test Result**	68	59	33
**Technical Requirement**	60–80	≥55	≥30
**Test Method**	T0604	T0606	T0605

This experiment uses steel slag produced by Panzhihua Iron and Steel Group. In order to ensure the stability of the steel slag, the aging time of the steel slag is more than 1 year. Various performance indicators of the steel slag used are tested, and the test results are shown in Table 3.

**Table 3:** Technical Properties of Steel Slag.

Technical Index	Test Result	Technical Requirement	Test Method
Crushing value/ %	16.1	≤ 26	T0316
Los Angeles abrasion value/ %	13.1	≤ 28	T0317
Polished Stone Value (PSV)	68	≥ 42	T0321
Apparent relative density/ g·cm ⁻ ³	3.328	≥ 2.60	—
Bulk relative density/ g·cm ⁻ ³	3.232	—	T0304
Flat and elongated particles/ %	10.9	≤ 15	T0312

SMA-13 asphalt mixture was used in the test. Steel slag aggregates with particle sizes of 9.5–16 mm and 4.75–9.5 mm were used to replace basalt coarse aggregates of the same size. The steel slag accounted for 0%, 50%, and 100% of the coarse aggregate volume, respectively. A dosage of 0% was used as the control group, and 100% represented full steel slag asphalt mixture. A medium replacement rate of 50% was selected as the intermediate gradient to facilitate the comparison of skid resistance evolution of mixtures at low, medium, and high dosages. Meanwhile, 50% dosage is a well-recognized optimal medium content in the literature [28]. Due to the large density difference between steel slag and basalt, volume control was adopted in the gradation design of mixtures. Based on the principle of equal volume replacement, the volumetric method was used to design the gradation composition of asphalt mixtures with different steel slag volume contents. The passing rate of each sieve size is shown in Table 4. The optimum asphalt-aggregate ratios of SMA-13 steel slag asphalt mixtures with the four dosages were 5.4%, 5.6%, 5.8%, 6.0%, and 6.2%, respectively.

**Table 4:** Passing Rates of Each Sieve Size.

Mix Type	Passing Percentage for Sieve Size (mm)									
0.075	0.15	0.3	0.6	1.18	2.36	4.75	9.5	13.2	16
Upper limit of gradation	12	15	16	20	24	26	34	75	100	100
Lower limit of gradation	8	9	10	12	14	15	20	50	90	100
Median gradation	10	12	13	16	19	20.5	27	62.5	95	100
SMA-13	10	11.7	13.2	15.7	19.9	23.9	30.2	67.6	90.8	100

### 2.2. Test methods

**Fig 1:**
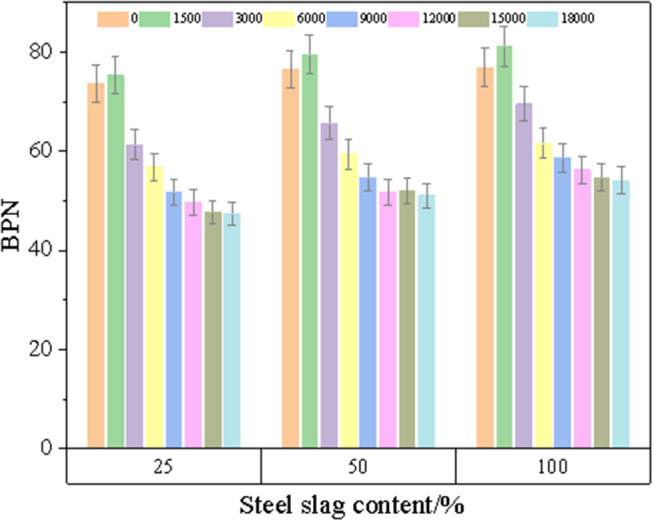
BPN values at different dosages and wear‌‌ cycles.

In accordance with test methods in the Field Test Regulations for Highway Subgrade and Pavement (JTG 3450−2019), SMA-13 asphalt mixture rutting plate specimens of 30030050 mm were prepared. Accelerated abrasion tests were performed using a self-developed small accelerated abrasion device, as shown in Fig 1. The wheel load in the accelerated loading test was 0.7 MPa. The abrasion apparatus was equipped with two rubber wheels with a width of 50 mm and a diameter of 200 mm, running at a frequency of 3000 r/h. Water was sprayed onto the specimens during the accelerated abrasion process.

Test temperatures were strictly controlled. Specimens were kept at a constant temperature of 20 °C, 40 °C, and 60 °C for 4 h to ensure uniform and stable internal temperatures. The accelerated abrasion device was equipped with a constant temperature chamber, which was heated and insulated by built-in heating modules and a circulating air system to keep the ambient temperature consistent with the target test temperature. Specimens were placed into the chamber and tested only after the device temperature was stabilized.

The British Pendulum Number (BPN) of the specimen surface was measured by a pendulum friction tester, and the Mean Texture Depth (MTD) was measured by the sand patch method. Three parallel samples were set in each test to control experimental errors. After the test, the three groups of parallel data were preprocessed first, and the data discreteness was verified by calculating the standard deviation. After confirming no abnormal dispersion, the average value of the three parallel tests was taken as the final test data.

## 3. Results and discussion

### 3.1. Skid-Resistance Indicators under Different Steel Slag Contents

**Fig 2:**
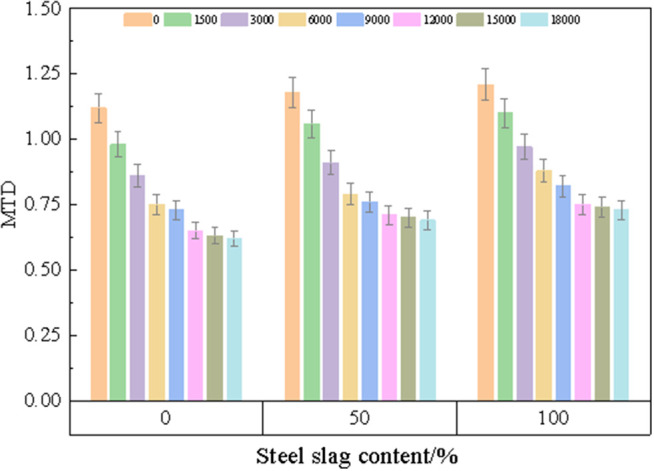
MTD values at different dosages and wear cycles.

To investigate the influence of steel slag content on the skid-resistance performance of SMA-13 asphalt mixtures, three types of rutting slab specimens were prepared with steel slag contents of 0%, 50%, and 100%. Accelerated abrasion tests were conducted using a laboratory accelerated wear device, and the BPN and MTD values at different abrasion cycles were measured. The test results are shown in Figs 1 and 2.

As shown in Fig 1, the BPN values of steel slag asphalt mixtures with different contents present the same overall trend. The skid resistance increases at the initial abrasion stage, then decreases rapidly and finally tends to be stable. Both the initial and final BPN values rise with the increase of steel slag content, and the difference between the initial value and the maximum value also increases. For example, the BPN value increases by 2.3% from the initial value to the maximum value at 0% steel slag content, by 3.9% at 50% steel slag content and by 5.5% at 100% steel slag content, indicating that the skid resistance of steel slag asphalt mixtures is better than that of basalt asphalt mixtures. It is considered that steel slag has better angularity, richer surface texture and favorable micro-texture than basalt, resulting in higher skid resistance. In addition, the attenuation degrees of skid resistance are different for mixtures with different steel slag contents. The BPN attenuation rate is 35% for basalt asphalt mixture with 0% steel slag content and 29% for full steel slag asphalt mixture with 100% steel slag content. It is speculated that this is mainly due to a series of mineral crystals formed during the cooling process of steel slag. The minerals in steel slag are well matched in hardness and provide synergistic wear resistance, which is not easy to produce uneven wear and leads to a higher polished stone value. Therefore, the skid resistance of steel slag asphalt mixtures decays more slowly.

As shown in Fig 2, the MTD of asphalt mixtures decreases with the increase of abrasion cycles and tends to be stable finally, and steel slag asphalt mixtures with different contents show similar trends. Both the initial and final values of MTD increase with the increase of steel slag content. The MTD attenuation rate is 39% for full steel slag asphalt mixture with 100% steel slag content, 41.2% for mixture with 50% steel slag content and 45% for basalt asphalt mixture. It can be seen that the addition of steel slag can significantly slow down the attenuation of MTD. It is analyzed that the MTD of asphalt mixtures with three contents is affected by their different void ratios. The void ratios of the three mixtures are 3.41%, 3.63% and 3.92% respectively. It can be found that the void ratio of asphalt mixtures increases gradually with the increase of steel slag content. The full steel slag asphalt mixture has the largest void ratio and the best skid resistance.

### 3.2. Skid-resistance indicators at different temperatures

**Fig 3:**
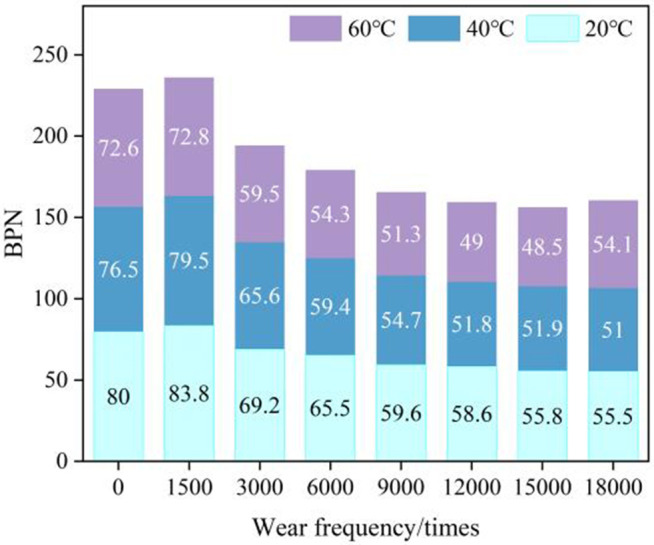
BPN values at different temperatures.

**Fig 4:**
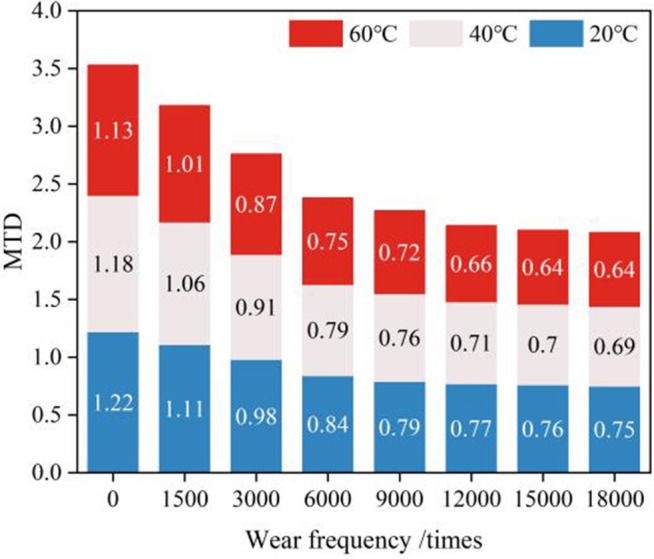
MTD values at different temperatures.

To investigate the effect of temperature on the skid resistance of asphalt mixtures with 50% steel slag content, this study selected three test conditions of 20°C, 40°C and 60°C. Laboratory accelerated abrasion tests were carried out on steel slag asphalt mixture rutting plate specimens at different temperatures. The BPN and MTD values of steel slag asphalt mixtures at different temperatures and abrasion cycles are shown in Fig 3 and Fig 4.

As shown in Fig 3, the figure presents the variation trend of BPN values of steel slag asphalt mixtures considering temperature effects. Overall, even under different temperature conditions, the BPN values of steel slag asphalt mixtures decrease with the increase of abrasion cycles. Both temperature gradient and abrasion cycle gradient are negatively correlated with BPN values, and the weakening effect of high temperature on BPN values is significant at all abrasion stages.

From a local quantitative perspective, when the temperature is 20°C and abrasion cycles increase from 0 to 18000, the BPN value decreases continuously from 80 to 55.5 with a total change of −24.5. The change rate differs obviously in different stages. The BPN value increases by 4.75% at 0−1500 cycles, which may be due to the uniform distribution of pavement micro-texture caused by initial abrasion and temporary optimization of skid resistance structure. The BPN value decreases continuously at 1500−18000 cycles with a total change rate of −33.8%. At 60°C, the change rate of initial difference is −9.2%, and the BPN value keeps decaying with the increase of abrasion cycles with a change rate of −31.0%. This phenomenon can be explained by high-temperature mechanical properties. High temperature enhances the viscoelasticity of steel slag asphalt mixtures, which makes the surface skid resistance texture easy to be damaged by abrasion and directly reduces the material friction coefficient. The skid resistance decreases greatly under the dual effects. The attenuation trend at 40°C is transitional between 20°C and 60°C. At 3000 abrasion cycles, the BPN value at 40°C decreases by 5.2% compared with that at 20°C and increases by 10.2% compared with that at 60°C, which further proves the gradual weakening effect of temperature on BPN values.

As shown in Fig 4, the figure presents the variation trend of MTD values of steel slag asphalt mixtures considering temperature effects. From the overall qualitative rule, MTD values are negatively correlated with temperature and abrasion cycles. Higher temperature and more abrasion cycles lead to more significant pavement texture depth loss. The weakening effect of high temperature (60°C) on texture depth is significant in all stages, and the initial texture depth reserve and abrasion resistance stability are better at low temperature (20°C).

At 20°C, the total change rate of MTD value is −38.5%, and the change rate differs obviously in different abrasion stages. The change rate is −19.7% at 0–3000 cycles and −23.5% at 3000–18000 cycles, indicating a relatively gentle loss rate of texture depth in the late abrasion stage.

At 60°C, the change rate of MTD initial value compared with that at 20°C is −7.4%, which indicates that high temperature has produced initial weakening on texture depth. The MTD value keeps decaying with the increase of abrasion cycles with a total change rate of −43.4%, and the change rate reaches −23.0% at 0–3000 cycles, reflecting the rapid loss of texture depth at the initial abrasion stage under high temperature.

The change at 40°C shows typical transitional characteristics. At 3000 abrasion cycles, the MTD value at 40°C decreases by 7.1% compared with that at 20°C and increases by 4.6% compared with that at 60°C, which further verifies the gradual weakening effect of temperature on texture depth. It is analyzed that the maintenance of pavement texture depth depends on the physical integrity of material micro-texture. The micro-convex bodies of steel slag asphalt mixture pavement are easy to be sheared and peeled off under high temperature (60°C) and abrasion, resulting in rapid loss of texture depth. At low temperature (20°C), the material has relatively high stiffness, stronger abrasion resistance of texture units and sufficient initial texture depth reserve. The correlation mechanism of temperature, mechanical properties and abrasion resistance is the core reason for the temperature gradient attenuation of MTD values.

### 3.3. Decay pattern of skid-resistance in steel-slag Asphalt Mixtures

To better fit the variation trends of BPN and MTD values of 50% content SMA-13 steel slag asphalt mixtures with abrasion cycles at different temperatures, this study sorts out common decay models adopted in research on road material performance degradation. Three typical models are selected for comparative verification with the YIdFert1 exponential decay model. They are listed as follows: ① Linear decay model (y = ax + b), applicable to uniform degradation processes; ② Power function decay model (y = ax^b^ + c), suitable for processes with nonlinear decay rate without obvious limit values; ③ Logarithmic decay model (y = a+blnx), applied to processes featuring fast initial decay and extremely slow later decay.

Multi-model fitting comparison is conducted on the abrasion attenuation data of BPN and MTD under different temperatures and steel slag contents. The results show that the coefficient of determination R^2^ of the YIdFert1 exponential decay model is higher than that of the other three models. The R^2^ values range from 0.75 to 0.88 for the linear model, 0.82 to 0.91 for the power function model, 0.78 to 0.89 for the logarithmic model, and 0.92 to 0.96 for the YIdFert1 model. Meanwhile, the residuals of the YIdFert1 model are closer to normal distribution with lower dispersion, which indicates its stronger capability in data interpretation. Combining fitting accuracy and physical mechanism adaptability, the YIdFert1 exponential decay model is finally adopted in this study.

**Fig 5:**
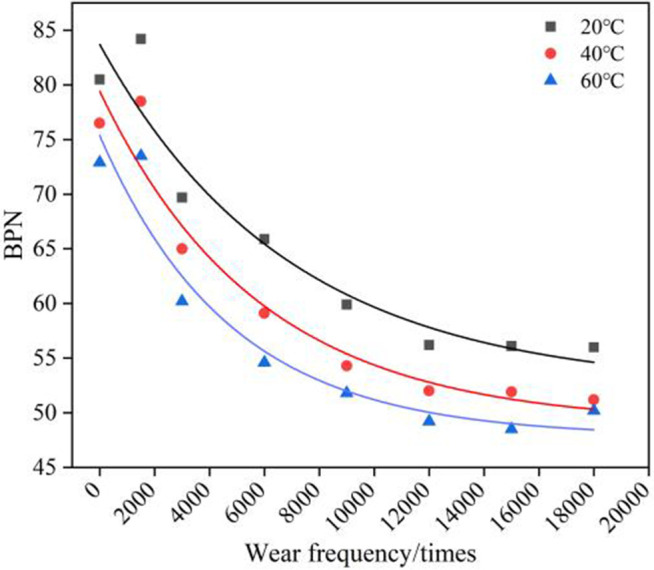
BPN value fitting.

**Fig 6:**
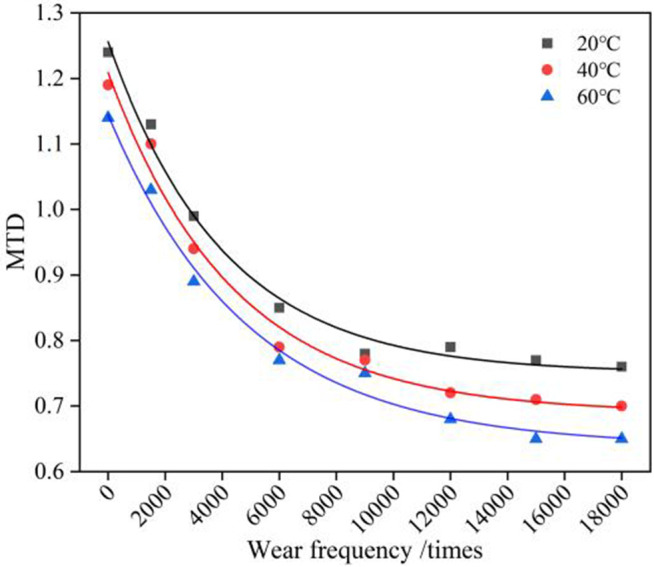
MTD value fitting.

Based on the above laboratory accelerated abrasion test results, the YIdFert1 model was used to fit the variation of BPN and MTD with abrasion cycles for SMA-13 steel-slag asphalt mixtures containing 50% steel slag at different temperatures. The YIdFert1 model is expressed as:y = a + be^-kx^

where *a* represents the final value after decay, *b* is the decay amplitude, and *k* is the decay rate. The fitted skid-resistance curves of SMA-13 steel-slag asphalt mixtures are shown in Figs 5 and 6.

As shown in Fig 5, the fitting curves indicate that the MTD of steel slag asphalt mixtures decreases significantly with the increase of abrasion cycles at different temperatures of 20°C, 40°C and 60°C. The attenuation rate of MTD becomes faster at higher temperatures, which suggests that a high-temperature environment accelerates the wear and degradation of the surface texture of steel slag asphalt mixtures. Meanwhile, the fitting correlation coefficients at all temperatures are above 0.96, demonstrating that the fitting model can accurately reflect the variation of MTD with abrasion cycles. It provides a reliable quantitative basis for studying the correlation between the abrasion resistance of steel slag asphalt mixtures and temperature, and also offers theoretical support for the evaluation and life prediction of texture depth durability in pavement engineering applications in different temperature regions.

As shown in Fig 6, the BPN values of steel slag asphalt mixtures under different temperatures all present a typical exponential decay trend with the increase of abrasion cycles, and the fitting correlation coefficients are all higher than 0.92. This indicates that the established decay model can well reflect the degradation law of surface skid resistance during the abrasion process. Overall, temperature has a significant effect on the attenuation of skid resistance. A high-temperature environment not only reduces the initial skid resistance level but also shortens the effective skid resistance life. Therefore, in pavement design for high-temperature areas, high-temperature resistant binder systems should be adopted preferentially, the gradation structure of steel slag particles should be optimized, and the surface texture depth should be appropriately increased to delay the attenuation process of skid resistance.

### 3.4. Correlation analysis

#### 3.4.1. Correlation analysis of BPN values.

**Fig 7:**
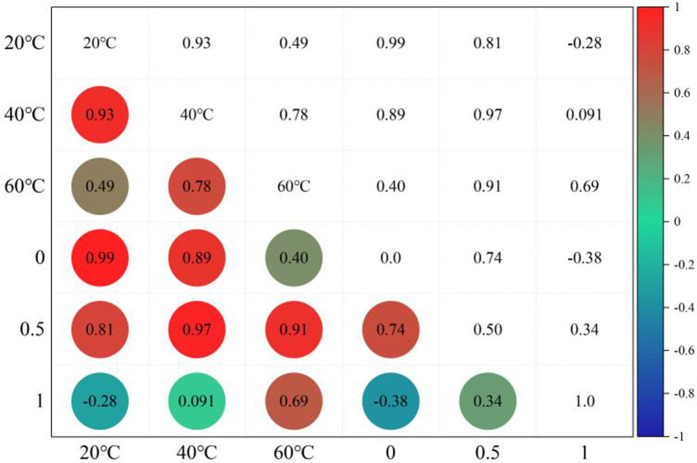
Correlation analysis of BPN under temperature and dosage factors.

**Fig 8:**
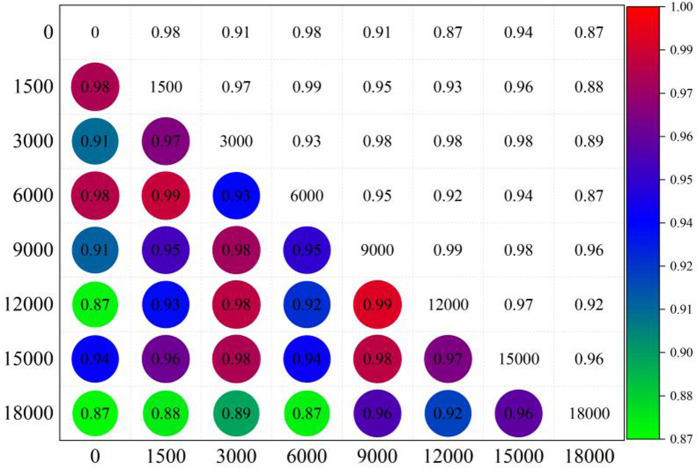
Correlation analysis of BPN under different wear cycles.

Different temperatures, steel slag contents and abrasion cycles all have influences on BPN. To further quantitatively describe the internal relationship of the above factors on BPN, correlation analyses were conducted for two factors including different temperatures and steel slag contents, and the results are shown in Fig 7. Correlation analyses were also carried out for different abrasion cycles, and the results are shown in Fig 8.

As shown in Fig 7, the correlation heatmap of BPN for steel slag asphalt mixtures under different temperatures and contents quantifies the interactive influence and internal correlation between temperature and steel slag content on BPN. In the temperature dimension, 20°C and 40°C show a strong positive correlation of 0.93, indicating that their influence rules on BPN are highly consistent. The correlation between 60°C and low temperature is significantly weakened, reflecting that the variation mechanism of friction coefficient of steel slag asphalt mixtures at high temperature is obviously different from that at medium and low temperatures. In the steel slag content dimension, 100% content and 50% content show a strong positive correlation, indicating that their enhancement effects on BPN are synergistic. The 0% content, namely pure basalt asphalt mixture, is negatively correlated with high steel slag content. It should be noted that correlation analysis can only reflect the covariation trend between variables and cannot reveal the internal logic of the action mechanism.

As shown in Fig 8, the correlation heatmap of BPN for steel slag asphalt mixtures under different abrasion cycles quantifies the internal correlation of BPN at different abrasion stages. From the perspective of color and value, a color closer to red indicates a stronger positive correlation, while a greener color indicates a relatively weaker correlation. Most correlation coefficients are above 0.87, and some even reach 0.99, showing that the variation rules of BPN under different abrasion cycles are highly consistent. From the analysis of abrasion stages, low abrasion cycles such as 0, 1500 and 3000 cycles show strong correlation of 0.98 and 0.97, indicating that the influence mechanism of initial abrasion on BPN is stable. With the increase of abrasion cycles, the correlation between 9000 cycles and 12000 cycles rises significantly to 0.99, reflecting that the variation rule of BPN in the later stage of abrasion is different from that in the early stage. This may be due to the aggravated wear of surface texture, which changes the attenuation mechanism of friction coefficient. It should be noted that correlation analysis can only reflect the covariation trend between variables and cannot reveal the internal logic of the action mechanism.

To further clarify the effects of temperature and steel slag content on the friction coefficient BPN of asphalt mixtures, two-way repeated measures analysis of variance was carried out on the test data with abrasion cycles as the repeated measurement factor. The results are shown in Table 5. Temperature has an extremely significant effect on BPN with an F value of 38.21 and a P value less than 0.001. The BPN value decreases with the increase of temperature. The average BPN value is about 68.3 at 20°C and about 58.9 at 60°C. Steel slag content also has an extremely significant effect on BPN with an F value of 20.82 and a P value less than 0.001. The average BPN value at 100% content is about 67.4, slightly higher than that at other contents. The interaction effect between temperature and steel slag content is not significant with an F value of 0.63 and a P value of 0.643, indicating that there is no significant interaction between the two factors on BPN.

**Table 5:** Results of Two-Way ANOVA for BPN Values.

Effect	Sum of squares（SS）	Degree of freedom（df）	Mean square（MS）	F-value	P-value
Temperature	1187.24	2	593.62	38.21	＜0.001
Dosage	646.76	2	323.38	20.82	＜0.001
Temperature and dosage interaction	38.86	4	9.72	0.63	0.643

#### 3.4.2. Correlation Analysis of MTD Values.

**Fig 9:**
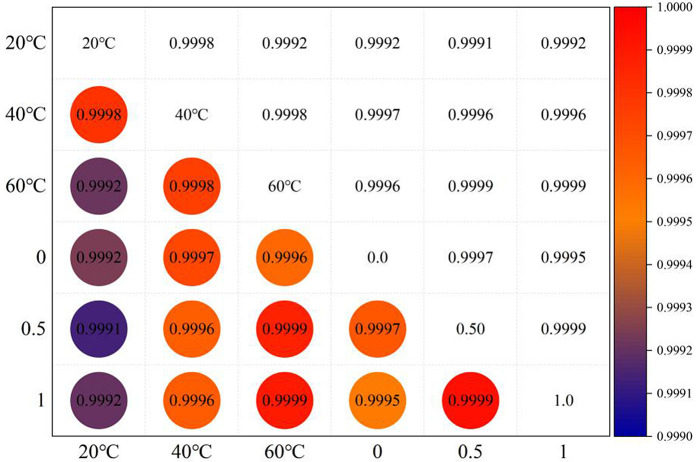
Correlation analysis of MTD under temperature and dosage factors.

**Fig 10:**
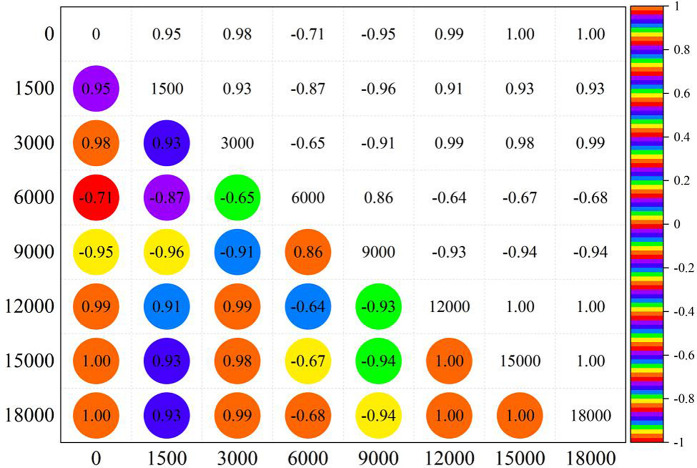
Correlation analysis of MTD under different wear cycles.

MTD is influenced by temperature, steel slag content, and abrasion cycles. To further quantify the intrinsic relationships of these factors with MTD, a correlation analysis was conducted for the two factors of temperature and steel slag content, with results shown in Fig 9. Additionally, a correlation analysis for different abrasion cycles was performed, with results presented in Fig 10.

As shown in Fig 9, the correlation heatmap of Mean Texture Depth (MTD) of steel slag asphalt mixtures under different temperatures and contents quantifies the interactive correlation between temperature and steel slag content on MTD. From the color and value characteristics, a color closer to red represents a stronger positive correlation. All correlation coefficients are above 0.99 except that the correlation between 50% content and 100% content is 0.50, indicating that the variation rules of MTD under different combinations of temperature and steel slag content are highly consistent. In the temperature dimension, the pairwise correlations among 20°C, 40°C and 60°C are all close to 1. For example, the correlation between 20°C and 40°C is 0.9998, showing that the influence of temperature on MTD is highly coordinated in trend. In the steel slag content dimension, 0%, 50% and 100% contents also show a strong positive correlation. For instance, the correlation between 0% and 100% content is 0.9995, reflecting that the modification effect of steel slag content on MTD has a stable linkage. For the special case that the correlation between 50% content and 100% content is 0.50, it is speculated that there may be staged differences in the mechanism of gradation and distribution of steel slag particles on texture depth within this content range, but the overall correlation is still relatively strong. It should be noted that correlation analysis can only reflect the covariation trend between variables and cannot reveal the internal logic of the action mechanism.

As shown in Fig 10, the correlation heatmap of Mean Texture Depth (MTD) of steel slag asphalt mixtures under different abrasion cycles clearly shows the correlation characteristics through colors and values. The low abrasion stages including 0, 1500 and 3000 cycles and the high abrasion stages including 12000, 15000 and 18000 cycles present strong positive correlation in pairwise comparison, reflecting the high consistency of MTD degradation mechanism in these two stages. The middle abrasion stages including 6000 and 9000 cycles show significant negative correlation with low and high abrasion stages, indicating the staged difference of MTD degradation mechanism during the abrasion process. It should be noted that correlation analysis can only reflect the covariation trend between variables and cannot reveal the internal logic of the action mechanism.

To clarify the effects of temperature and steel slag content on the MTD of asphalt mixtures, two-way repeated-measures analysis of variance was performed on the test data with abrasion cycle taken as the repeated measurement factor, and the results are listed in Table 6. Temperature exhibits an extremely significant influence on MTD with an F value of 12.71 and a P value less than 0.001, and higher temperature corresponds to lower MTD, with the average MTD of approximately 0.92 at 20°C and about 0.85 at 60°C. Steel slag content has a relatively significant effect on MTD with an F value of 4.02 and a P value of 0.023, and the average MTD reaches around 0.90 at the steel slag content of 50%, which is slightly higher than those at other contents. The interaction effect between temperature and steel slag content is insignificant with an F value of 0.84 and a P value of 0.03, indicating that there is no obvious interactive coupling effect of the‌‌ two factors on MTD.

**Table 6:** Results of Two-Way ANOVA for MTD Values.

Effect	Sum of squares（SS）	Degree of freedom（df）	Mean square（MS）	F-value	P-value
Temperature	2.038	2	1.019	12.71	＜0.001
Dosage	0.644	2	0.322	4.02	0.023
Temperature and dosage interaction	0.401	4	0.100	1.25	0.298

## 4. Conclusions

(1) Steel slag content exerts a significant regulatory effect on the skid resistance attenuation of asphalt mixtures, and its skid durability is much better than that of ordinary basalt asphalt mixtures. This finding provides a core basis for the selection of pavement skid-resistant aggregates and content design. Further research will be conducted to explore the internal correlation between micro-texture evolution and macro skid resistance attenuation, so as to further improve the mechanism analysis system.
(2) The deterioration effect of temperature on the skid resistance of steel slag asphalt mixtures presents a continuous gradient characteristic and shows obvious dependence on abrasion stages. In engineering application scenarios with high temperature, heavy load and large traffic volume, steel slag asphalt mixtures with high content are recommended to offset the coupled deterioration caused by high temperature and long-term abrasion. For projects with medium and low temperature and moderate traffic volume, steel slag asphalt mixtures with medium and low content can be selected considering economic benefits, which balances skid resistance and the resource utilization efficiency of steel slag solid waste.
(3) Both BPN and MTD of steel slag asphalt mixtures at different temperatures follow the YIdFert1 exponential attenuation law, and all correlation coefficients exceed 0.92. High temperature accelerates texture damage and texture depth loss, and greatly shortens the pavement skid resistance service life. The established model can characterize the skid decay law of mixtures with high accuracy, and provide a reliable quantitative basis for pavement skid design and material optimization in different climate zones.
(4) Temperature, steel slag content and abrasion process have significant and quantifiable influences on the skid resistance of steel slag asphalt mixtures. Both BPN and MTD exhibit staged degradation characteristics. Mechanism differentiation of BPN appears under elevated temperature and high steel slag content, while MTD shows an obvious turning point in the middle abrasion stage. The middle abrasion stage shall be taken as the key maintenance node for skid resistance in pavement maintenance design. There is no significant coupling effect of temperature and steel slag content on the two skid resistance indicators. It means that the optimization schemes for temperature and content can be designed separately, and performance fluctuations caused by their coupling effect can be‌‌ ignored.

## Supporting information

S1 Data Supporting information. (XLSX)
